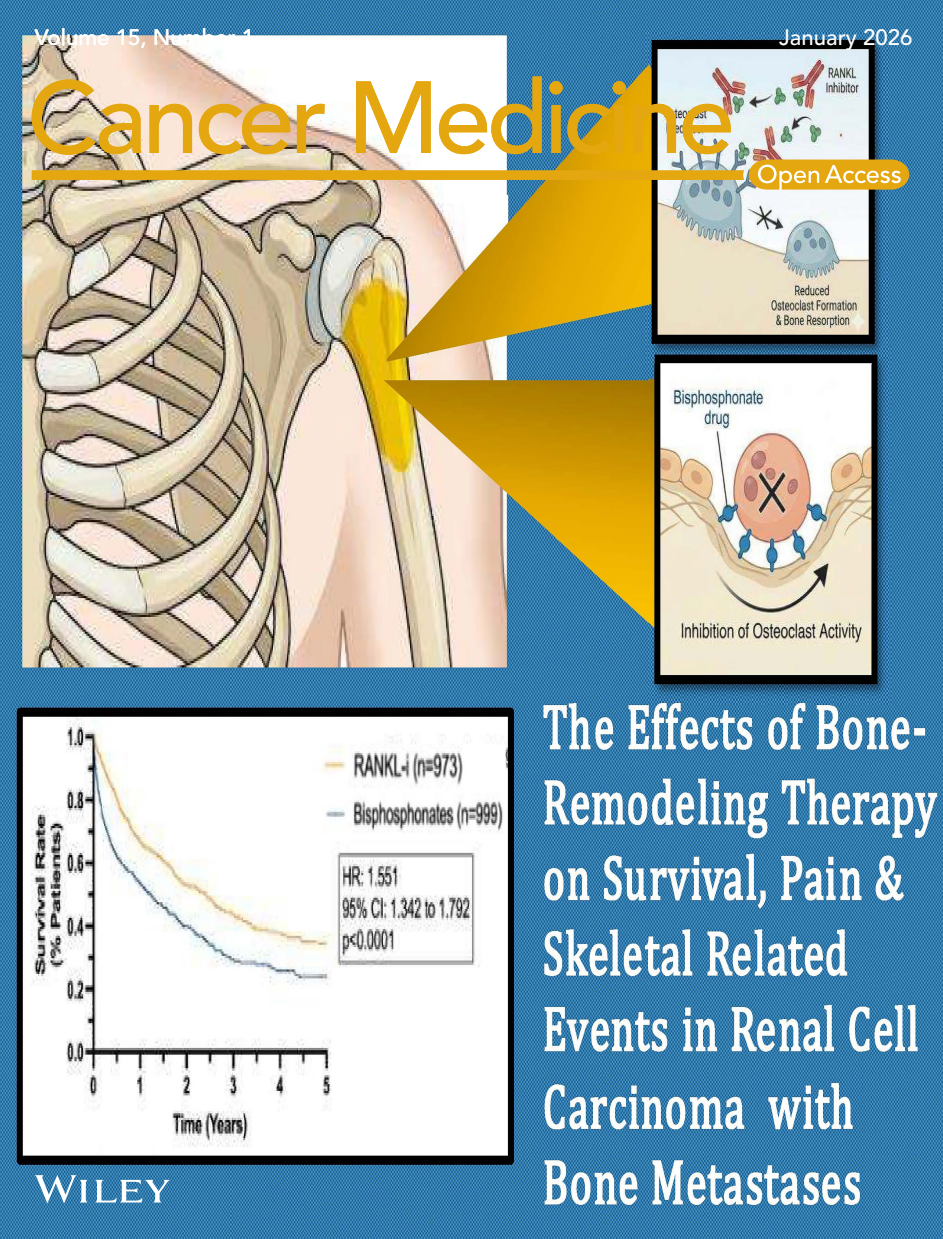# Cover Image

**DOI:** 10.1002/cam4.71632

**Published:** 2026-02-23

**Authors:** Brian H. Im, Kevin K. Zarrabi, Aaron R. Hochberg, Mihir S. Shah, James R. Mark, Joseph K. Izes, Patrick T. Gomella, Costas D. Lallas, Leonard G. Gomella, Adam R. Metwalli

## Abstract

The cover image is based on the article *The Effects of Bone‐Remodeling Therapy on Survival, Pain, and Skeletal Related Events in the Setting of Renal Cell Carcinoma With Bone Metastases: A Multicenter Investigation From a Large Global Health Research Network (TriNetX)* by Brian H. Im et al., https://doi.org/10.1002/cam4.71133.